# Apoptosis Gene Hunting Using Retroviral Expression Cloning: Identification of Vacuolar ATPase Subunit E

**DOI:** 10.1100/tsw.2003.07

**Published:** 2003-03-17

**Authors:** Claire L. Anderson, Gwyn T. Williams

**Affiliations:** School of Life Sciences, Keele University, Keele, Staffordshire, ST5 5BG, England

**Keywords:** vacuolar ATPase (adenosinetriphosphatase) subunit E, retroviral expression cloning, apoptosis/programmed cell death, pH, intracellular acidification, interleukin-3 (IL-3)

## Abstract

Over the past 10-15 years there has been an explosion of interest in apoptosis. The delayed realisation that cell death is an essential part of life for any multicellular organism has meant that, despite the recent and rapid developments of the last decade, the precise biochemical pathways involved in apoptosis remain incomplete and potentially novel genes may, as yet, remain undiscovered. The hunt is therefore on to bridge the remaining gaps in our knowledge. Our contribution to this research effort utilises a functional cloning approach to isolate important regulatory genes involved in apoptosis. This mini-review focuses on the use and advantages of a retroviral expression cloning strategy and describes the isolation and identification of one such potential apoptosis regulatory gene, namely that encoding vacuolar ATPase subunit E.

## INTRODUCTION

### Apoptosis Gene Hunting Using Retroviral Expression Cloning cDNA expression cloning is a powerful technique for the isolation of genes which confer selectable phenotypes on specific cell types. Numerous reports highlight the success of this approach and include the identification of potential oncogenes[1], genes capable of suppressing cytokine signal transduction[2], genes encoding cell surface antigens[3], and cDNAs able to confer autonomous growth to factor-dependent FCD-P1 cells[4,5]. We have utilised a functional expression cloning strategy using a retroviral vector, pRUF*neo*[4], to isolate apoptosis-controlling genes, employing a system developed by Gonda and colleagues[2,3,4]. One invaluable advantage of this functional approach is that it assumes no prior knowledge of involvement in apoptosis, i.e., it is completely unbiased and makes no assumptions concerning nucleotide sequence or protein-protein interactions. As a consequence, novel targets and unpredicted mechanisms may be discovered.

There are a number of advantages associated with the use of retroviral expression systems. Firstly, retroviral gene delivery is generally faster, more reliable, and has broader utility than alternative gene transfer methods for suspension cell cultures. The efficiency of virus-mediated gene transfer (infection) is also much higher than that of standard transfection methods, reliably leading to infection efficiencies exceeding 90%. This therefore enables the transfer and functional screening of very large libraries[1]. In addition, the viral DNA stably integrates into target cells in a predictable configuration, in the majority of cases at one copy per cell. This allows for expansion of individual infected cells displaying a particular phenotype and facilitates recovery of sequences inserted in the provirus[4]. Finally, in addition to its reliability, retroviral infection is one of the easiest laboratory techniques to use and simply involves the addition of virus-containing media to dividing target cells.

## Experimental Strategy

The functional screening system comprised a retroviral cDNA library, generated from the murine factor-dependent FDC-P1 cell line[2] as the source of apoptosis regulators, the murine BAF-3 pro-B lymphocyte cell line[6] as the screening host, and IL-3 deprivation as the apoptotic stimulus. The experimental strategy is based on the assumption that retroviral infection of BAF-3 cells with a cDNA library will allow a subset of cells to survive apoptosis following growth factor (IL-3) deprivation. Thus, using this directionally cloned cDNA library, selection of sense cDNAs able to suppress this apoptotic stimulus could reflect the expression of a functional copy, e.g., the complete open reading frame, of a protein with antiapoptotic function, or a partial open reading frame generating either loss-of-function of a proapoptotic gene or gain-of-function of an antiapoptotic gene.

Library construction, cloning, amplification, and subsequent infection into target cells was conducted essentially as described[4]. BAF-3 cells, infected with retroviral vector (pRUF*neo*) containing the cDNA library, were subjected to IL-3 deprivation and the surviving population of cells cloned in soft agar to generate IL-3 resistant clones (Figs. 1 and 2). Genomic DNA isolated from IL-3 resistant clones was used in a PCR-based approach utilising retroviral vector primers[4] to rescue cDNA inserts conferring the resistant phenotype, which were subsequently identified by sequencing.

## Identification of Vacuolar ATPase Subunit E

This functional expression cloning approach to apoptosis gene hunting has lead to the isolation and identification of several potentially significant genes/gene fragments involved in IL-3 deprivation-induced apoptosis in BAF-3 cells[7]. One of the most interesting cDNAs isolated from this study showed high identity (98%) to the 3’ untranslated region (UTR) of the gene encoding vacuolar ATPase (v-ATPase) subunit E.

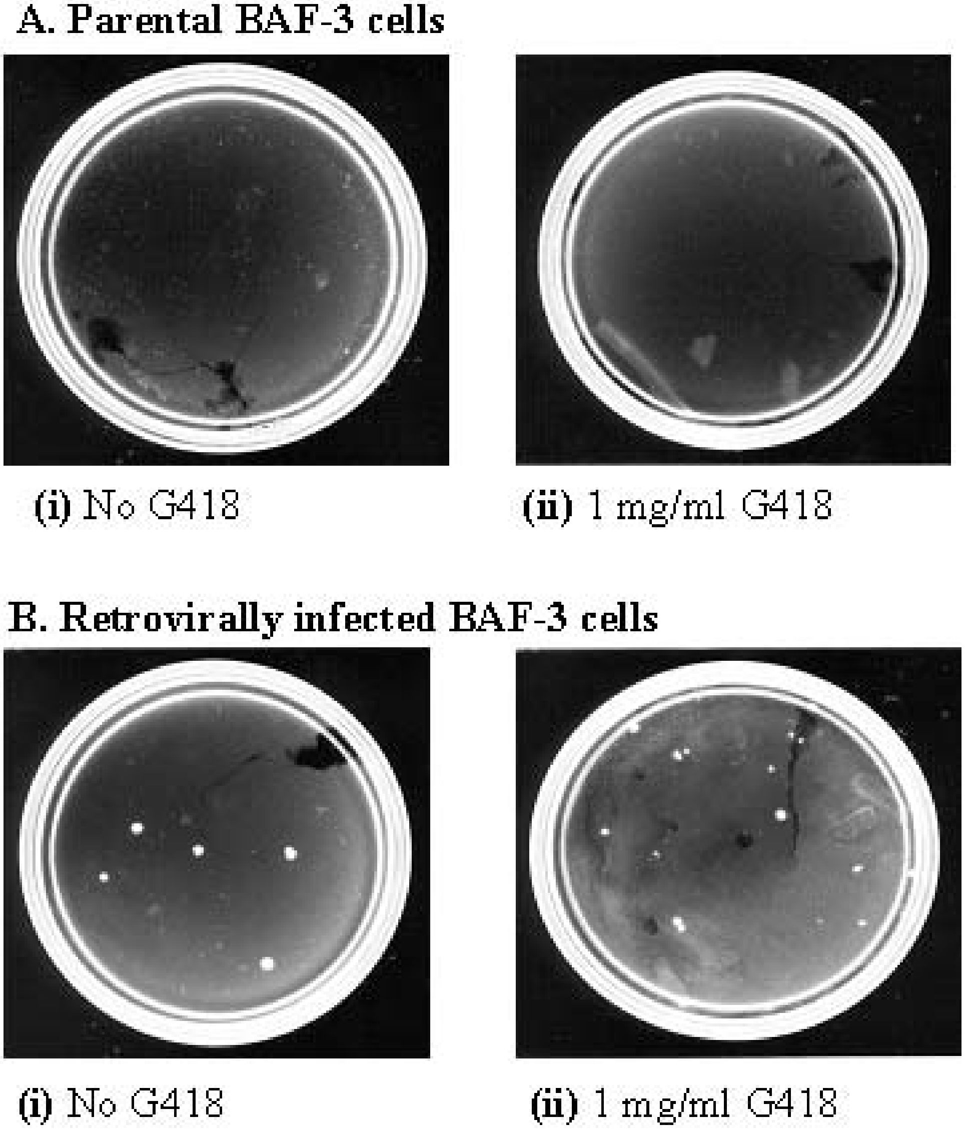

**FIGURE 1.** Colony forming ability of parental and retrovirally infected BAF-3 cells following IL-3 deprivation-induced apoptosis. Parental and retrovirally infected BAF-3 cells were subjected to 120 h IL-3 deprivation and cloned in soft agar in the presence of the indicated concentrations of G418 ($1.5 \times 10^7$ total cells plated/dish). Levels of spontaneous IL-3 resistance in parental BAF-3 cells were nil at all time points examined, independent of G418 concentration, indicative that integration of cDNA from the expression library into host cells was likely responsible for the observed resistant phenotype of retrovirally infected cells. Representative plates are illustrated.

## Structure and Function of v-ATPases

The v-ATPase is a fundamentally important enzyme[8]. In almost every eukaryotic cell, this highly conserved proton pump couples hydrolysis of cytosolic ATP to proton transport, leading to acidification of a wide variety of organelles and membranes. These include clathrin-coated vesicles, endosomes, lysosomes, Golgi, secretory vesicles, and the central vacuoles of plants and lower eukaryotes [as reviewed in 8,9,10,11,12,13]. Acidification of vacuolar compartments plays an important role in processes such as receptor-mediated endocytosis[14,15], intracellular

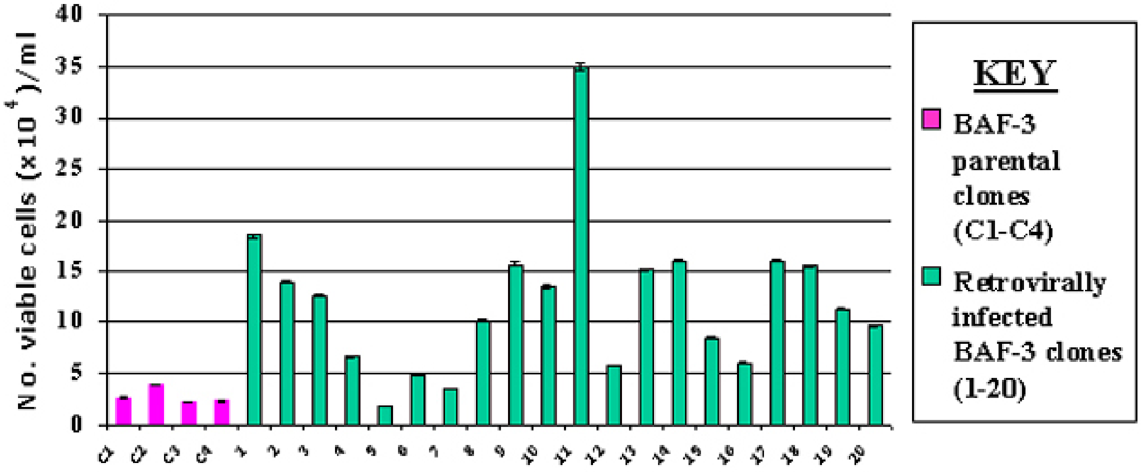

**FIGURE 2.** Confirmation of IL-3 resistant phenotype of retrovirally infected BAF-3 clones. IL-3 resistant retrovirally infected BAF-3 clones were repeatedly washed (3 times) in RPMI medium to remove IL-3 and cells seeded at $2 \times 10^5$ cells/ml in medium devoid of IL-3. Cell viability was determined by vital dye exclusion at 48 h post-IL-3 deprivation. Parental BAF-3 clones (C1-C4) derived from cells cloned in soft agar in the presence of IL-3, were subjected to the same treatment and acted as controls to determine basal levels of IL-3 resistance. Results shown are means ± S.E. of triplicate samples for a selection of clones. Representative data are illustrated.

membrane trafficking[16,17], protein processing[18] and degradation[19], and the coupled transport of small molecules[20]. In addition, v-ATPases also play an important role in the plasma membrane of various specialized cells. Of particular interest are the v-ATPases of macrophages and neutrophils, which assist in pH homeostasis[21,22] and the ability of tumour cells to target v-ATPases to the plasma membrane creating an acidic extracellular environment necessary for invasion and metastasis[23]. Finally, and most significantly in the context of our research, v-ATPase expression has also been implicated in apoptosis[24,25,26,27,28,29].

In terms of structure, v-ATPases are multisubunit complexes composed of two functional domains, $V_1$ and $V_0$. The peripheral (cytoplasmic-facing) $V_1$ domain, a 570-kDa complex composed of eight subunits (denoted A-H and including the E subunit) ranging in molecular weight 70–13 kDa, is responsible for ATP hydrolysis. The integral (membrane-bound) $V_0$ domain is a 260-kDa complex composed of five subunits (denoted a, d, c, c′ and c′′) of molecular weight 100–17 kDa that is responsible for proton translocation across the membrane[9]. ATP-driven proton transport requires structural and functional coupling of these two domains, and *in vivo*, the interaction between $V_1$ and $V_0$ is dynamic and regulated by extracellular conditions[30]. The v-ATPases appear to contain both catalytic and noncatalytic nucleotide binding sites, with the catalytic sites residing on the 70-kDa A subunit and the noncatalytic sites located on the 60-kDa B subunit[11]. The function of these noncatalytic sites remains uncertain, although recent observations suggest that occupancy of these sites may be required for maximal v-ATPase activity[31]. The remaining accessory $V_1$ subunits — (C-H) including subunit E — play an important, although as yet undefined, role in v-ATPase activity.

## Structure and Predicted Function of v-ATPase Subunit E

The v-ATPase subunit E gene was initially cloned from bovine kidney[32] and then subsequently from yeast[33] and designated *VMA4*[34]. Studies in yeast have proved a powerful tool for the identification of genes encoding v-ATPase subunits (*VMA* genes) because of the conditional lethal phenotype observed upon their disruption[12,]. Indeed, disruption of the *VMA4* gene produced a phenotype identical to other null mutants and established its essential role in $V_1$ assembly[34,35]. In addition, the cloning and expression of human subunit E[36] and the first invertebrate v-ATPase subunit E from the model insect, *Manduca sexta,* has also been reported[37].

The predicted structure of the E subunit contains elongated $\alpha$-helices, which has lead to speculation that it constitutes part of the stalk architecture of the v-ATPase[9,38]. In addition, this $\alpha$-helical structure resembles that of the $F_1$ $\gamma$ subunit, the central component of the $F_1$ rotor and thus the E subunit may play a similar role to $\gamma$ in the v-ATPases. Although the mechanism by which v-ATPases carry out ATP-dependent proton translocation has not been established, it is thought to be similar to the rotary mechanism recently proposed for F-ATPases, whereby hydrolysis of ATP forces rotation of the central $\gamma$ subunit ultimately leading to movement of protons across the membrane[8,9].

The specific function of v-ATPase subunit E has yet to be ascertained, although results from yeast mutants[33,34] and immunological evidence regarding differential expression of subunit E isoforms in mammalian kidney[39], argue in favour of a role in enzyme biogenesis and/or targeting. In addition, the E subunit can crosslink to the c subunit of the $V_0$ domain, suggesting that it may play a role in attachment and coupling of the $V_1$ and $V_0$ domains[9,40].

## Mechanism of v-ATPases

One of the central questions concerning v-ATPases involves the mechanism by which cells are able to regulate vacuolar acidification *in vivo*. It is clear that cells can control the pH of different intracellular compartments independently and a number of mechanisms to control vacuolar pH have been postulated. These include regulation via reversible dissociation of $V_1$ and $V_0$ domains[30], reversible disulfide bond formation between cysteine residues at the catalytic site[41], and changes in coupling efficiency between proton transport and ATP hydrolysis[42]. In addition, control of counter-ion (chloride) conductance[9] and the influence of activator[43] and inhibitor proteins[44] on v-ATPase activity may also function to regulate vacuolar pH. In view of the diversity of functions of v-ATPases, cells are most likely to employ multiple mechanisms for controlling their activity[11].

## Apoptosis and v-ATPase

The mechanism by which the v-ATPase subunit E gene is capable of apoptotic repression remains unclear, particularly in view of the fact that the v-ATPase is a multifunctional, multisubunit enzyme and that only 338 bp of 3' UTR sequence from one such subunit was isolated from this study. In light of the function of the v-ATPase proton pump in the acidification of a wide variety of organelles and membranes, a speculative mechanism to explain its role in apoptotic suppression may involve the modulation of intracellular pH. There is accumulating evidence that a fall in intracellular pH (pHi) occurs in cells which are committed to undergo apoptosis following growth factor deprivation[28,45,46,47,48]. Emerging supportive evidence also suggests that an alteration in cellular pH regulation represents an early event associated with apoptosis via the mitochondria-dependent or intrinsic cell death pathway [as reviewed in 49,50].

## CONCLUSION

This mini-review has described a gene hunting strategy utilising retroviral expression cloning designed to isolate important, potentially novel regulatory genes involved in apoptosis initiated by IL-3 deprivation. This functional screening system incorporated a mouse cDNA library, generated from the factor-dependent FDC-P1 cell line, as the source of apoptosis regulators, the murine BAF-3 pro-B lymphocyte cell line as the screening host, and IL-3 withdrawal as the apoptotic stimulus. The experimental strategy is based on the assumption that retroviral infection of BAF-3 cells with a cDNA library will allow a subset of cells to survive apoptosis induced by growth factor (IL-3) deprivation. As the cDNA library was directionally cloned, selection of sense cDNAs able to suppress this apoptotic stimulus should reflect the expression of either the complete open reading frame of a protein with antiapoptotic function, or a partial open reading frame generating either loss-of-function of a proapoptotic gene or gain-of-function of an antiapoptotic gene.

The utilisation of a functional expression cloning strategy using the retroviral vector, pRUF*neo,* has lead to the isolation of several potentially significant genes/gene fragments involved in IL-3 withdrawal-induced apoptosis in BAF-3 cells. One such cDNA insert described displays high identity to the gene encoding vacuolar ATPase (v-ATPase) subunit E. Further experimental data is required to verify the potential importance of the vacuolar subunit E gene in the suppression of IL-3 deprivation-induced apoptosis and determine its mechanism of action.

## ACKNOWLEDGEMENTS

This work was supported by a Leukaemia Research Fund grant (to C.L.A.) and the Wellcome Trust (to G.T.W.). The cDNA library was kindly donated by Thomas Gonda and colleagues, Hanson Institute, Adelaide, Australia. We also acknowledge and thank our colleagues at the Walter and Elisa Hall Institute, Melbourne, Kings College Hospital Medical School, London and at the School of Life Sciences, Keele University for valuable help and assistance.
